# Postoperative Opioid Use After Bariatric Surgery and Associated Factors: A Retrospective Comparison of Laparoscopic and Robotic Approaches

**DOI:** 10.3390/jcm15145705

**Published:** 2026-07-21

**Authors:** Maryam Hassanesfahani, Dimitrios Giannis, Ruby Zhao, Patrick Kiarie, Hiranya S, Armon Farhani, Benjamin Hershfeld, Luke Keating, Martine Louis, Noman Khan, Darshak Shah

**Affiliations:** 1 Department of General Surgery, Flushing Hospital Medical Center, Flushing, Queens, NY 11355, USA; ruby.zhao94@gmail.com (R.Z.); patrickkiarie70@gmail.com (P.K.); afarhani@nyit.edu (A.F.); hershfeldbenjamin@gmail.com (B.H.); mlouis2@jhmc.org (M.L.); nomisurgery@gmail.com (N.K.);; 2 Department of Research, Education and Innovation, MediSys Health Network, Flushing, Queens, NY 11355, USA; luke.h.keating@gmail.com

**Keywords:** bariatric surgery, robotic surgery, psychiatric history, opioid use, minimally invasive surgery

## Abstract

**Background/Objectives**: The opioid epidemic has increased awareness of inpatient opioid use after surgery. Effective pain control is essential for recovery; however, factors influencing postoperative opioid requirements (PORs) remain unclear. This study examined patient-specific factors and surgical platforms associated with postoperative rescue opioid use within 48 h of bariatric surgery. **Methods**: A retrospective chart review was conducted on patients undergoing bariatric surgery (Roux-en-Y gastric bypass (RYGB) or sleeve gastrectomy (SG)) from March 2021 to March 2023 at a community hospital setting. Data included demographics, comorbidities, psychiatric history, prior surgeries (cholecystectomy, hernia repair, other bariatric surgery), intraoperative parameters, postoperative disposition, hospital length of stay, and PORs measured in morphine milligram equivalents (MME). Robotic and laparoscopic surgery were compared and risk factors for increased PORs were analyzed. **Results**: Among 442 patients (86.7% women, mean age 42.1 ± 12.0 years), robotic procedures (51.6%) had higher PORs than laparoscopic procedures (13.3 ± 13.2 versus 10.1 ± 11.2 MME; *p* < 0.01), mainly driven by the SG group (14.8 ± 13.3 versus 9.2 ± 9.6 MME, respectively; *p* < 0.01). In multivariable logistic regression, robotic surgery (OR 1.38, 95% CI: 1.02–1.88, *p* = 0.041), psychiatric history (OR 1.67, 95% CI: 1.11–2.51, *p* = 0.014), and prior surgery (OR 1.52, 95% CI: 1.03–2.24, *p* = 0.034) were associated with increased PORs (>10 MME). No significant differences were observed based on sex, type of procedure (SG vs. RYGB), or body mass index. **Conclusions**: In this study, robotic surgery was associated with higher PORs. Preoperative psychiatric evaluation and multimodal pain management strategies may assist in the optimization of analgesia in patients with psychiatric conditions or prior surgery.

## 1. Introduction

Inpatient opioid use in the postoperative period has received increased attention as the opioid epidemic remains one of the most pressing public health crises [1]. Effective pain control is crucial for both faster recovery and patient comfort. Pain threshold and medication requirements are determined by various factors, including the type, extent and duration of surgery, the anesthesia technique (local and/or general), intraoperative pain management, and patient factors [2,3]. After bariatric surgery, permanent anatomical alterations affect oral analgesic absorption. The small gastric pouch of Roux-en-Y gastric bypass (RYGB) or the narrow elongated stomach of sleeve gastrectomy (SG) accelerates gastric emptying. Oral morphine 30 mg after RYGB showed a two-fold reduction in time to maximum concentration and a 1.7 times increase in maximum concentration between preoperative assessment and 2 weeks postoperatively in the study by Lloret-Linares et al. [4]. SG accelerates liquid and solid gastric emptying and small intestinal transit, and increases bile acid serum levels, while bypass of the duodenum and proximal jejunum in RYGB reduces absorptive surface area and delays mixing with bile salts, impairing dissolution of lipophilic drugs [5,6]. Accelerated orocecal transit further limits intestinal residence time, potentially rendering extended-release formulations ineffective [6]. In addition, the avoidance of long-term NSAIDs after RYGB (due to marginal ulcer risk) eliminates a key opioid-sparing analgesic and may indirectly increase opioid requirements [7,8]. Overall, these changes make oral analgesic bioavailability highly unpredictable, necessitating individualized dosing strategies [9,10]. Patients have a wide range of opioid use even after the same procedure, suggesting that the variability in postoperative analgesic requirements is determined by perioperative factors as well as biopsychosocial patient characteristics [11,12]. A recent systematic review and meta-analysis of 39 studies encompassing over 294,000 patients showed that younger age, chronic pain, smoking, BMI out of normal range, anxiety, and preoperative opioid use were associated with increased pain and opioid use within the first 24 h after surgery [3]. Preoperative depression, substance use disorders, and pain catastrophizing have been identified as independent risk factors for increased postoperative opioid requirements and transition to persistent postoperative opioid use [12,13,14]. The American Society for Enhanced Recovery and Perioperative Quality Initiative joint consensus statement highlights that preoperative opioid use, depression, and substance use disorders are risk factors for persistent postoperative opioid use [13]. Understanding these individual determinants of opioid use in the early postoperative period may allow clinicians to better tailor analgesic regimens, set appropriate patient expectations, and ultimately reduce opioid-related adverse events without compromising pain control [1,15]. Several approaches have been proposed for effective pain management, such as multimodal pain control, nerve blocks, and opioid-sparing enhanced recovery protocols [16,17]. Further, minimally invasive surgery is associated with less pain and lower opioid requirements in the perioperative period [18,19,20]. Currently, there is a paucity of high-quality data comparing opioid requirements between laparoscopic and robotic approaches. This study aimed to explore the differences in postoperative rescue opioid use between laparoscopic and robotic bariatric surgery and to identify patient characteristics associated with increased opioid requirements.

## 2. Materials and Methods

This retrospective study included all adult patients (>18 years old) who underwent minimally invasive robotic-assisted or laparoscopic bariatric surgery (SG or RYGB) from March 2021 to March 2023 at a community hospital setting in the New York area. Institutional IRB approval was granted retrospectively with a waiver of informed consent for deidentified data on 20 September 2023 under study number 2029617-3. Epic System Inc.’s (Verona, WI, USA) SlicerDicer application (Epic version 09/2023) was used to extract the records of eligible patients and variables of interest were automatically or manually extracted by five authors (RZ, PK, HS, AF, and BH). Data abstractors independently reviewed the electronic medical records to manually verify extracted data and complete missing or non-standardized variables. To ensure data accuracy and consistency, each record was reviewed by at least two independent reviewers. Any discrepancies between abstracted datasets were flagged and resolved through consensus discussion among the extracting authors. If consensus could not be reached, a senior author served as adjudicator to make the final determination. Data cleaning and final dataset reconciliation were performed prior to statistical analysis.

At our institution, a bariatric protocol pathway is implemented in a standardized approach, including routine bilateral transversus abdominis plane (TAP) blocks performed at the conclusion of every procedure, multimodal analgesia with scheduled intravenous acetaminophen and NSAIDs postoperatively for 24 h (day 0) when not contraindicated, and transition to oral form of these medications on day 1 after surgery; other adjuncts such as gabapentin, and lidocaine patches are uniformly applied across all patients. Opioids are given as needed for pain not controlled with other medications. All patients are discharged with a standardized opioid-free analgesic prescription of acetaminophen at the time of hospital discharge.

Data of interest included demographics (age, sex, body mass index (BMI)), comorbidities (diabetes mellitus (DM), hypertension (HTN), asthma, obstructive sleep apnea (OSA), gastroesophageal reflux disease (GERD), hiatal hernia (HH) Helicobacter pylori infection, osteoarthritis (OA), Charlson Comorbidity Index (CCI)), prior surgical history (cholecystectomy, hernia repair, other bariatric surgery), psychiatric history, intraoperative parameters (procedure type, procedure duration, postoperative location (telemetry unit versus intensive care unit (ICU)), hospital length of stay (LOS), and postoperative opioids (morphine, oxycodone, hydromorphone, fentanyl).

Continuous variables were described with means and standard deviations (SD) or medians and quartile ranges (25th quartile (Q1)–75th quartile (Q3)), depending on the shape of the variable distribution. Categorical variables were described using percentages and frequencies. Statistical tests (chi-square test for categorical variables, and *t*-test or Mann–Whitney U test for continuous variables) were conducted as appropriate to compare variables of interest between groups and included corresponding *p*-values. Missing data were minimal (<10% across all variables) and were handled using imputation.

Statistical analyses, including multivariable regression, were conducted to compare the total postoperative opioid consumption, expressed in morphine milligram equivalents (MME), between robotic and laparoscopic approaches, and to assess potential risk factors associated with increased postoperative analgesic requirements. The conversion of opioid doses to MME was performed using standardized conversion factors consistent with CDC MME tables [21]. Multivariable logistic regression analysis was conducted to adjust for confounding risk factors with odds ratios (ORs), confidence intervals (CIs), and *p*-values for each predictor. For multivariable logistic regression analysis, postoperative opioid consumption was dichotomized using a predefined threshold of 10 MME administered during the first 48 postoperative hours or prior to discharge, whichever was earlier. The 48 h window was selected to capture postoperative inpatient analgesic requirements, defined as a standardized observation period across variable LOS prior to discharge. Patients receiving >10 MME were classified as having high postoperative opioid requirements, whereas those receiving ≤10 MME were classified as having low postoperative opioid requirements. This threshold was selected to distinguish patients requiring greater postoperative rescue opioid analgesia and was used as the dependent variable in the regression model. A cutoff of 10 MME was selected a priori to identify patients requiring clinically meaningful postoperative rescue opioid analgesia. This threshold approximates the opioid requirement associated with multiple rescue doses during the early postoperative period and provides a clinically interpretable distinction between lower and higher inpatient opioid utilization while maintaining adequate numbers of patients in each group for multivariable analysis.

A value of *p* < 0.05 was considered statistically significant. All statistical analyses were performed using R version 4.2.3 within RStudio version 2023.03.0+386.

## 3. Results

### 3.1. Population Characteristics

The study sample included 442 patients, mainly females (86.7%), with a mean age of 42.1 ± 12.0 years (Table 1).

Comorbidities included DM in 37.3%, HTN in 46.8%, asthma in 27.4%, OSA in 80.5%, GERD in 31.0%, hiatal hernia in 30.1%, H. pylori infection in 14.5%, OA in 12.4%, and psychiatric conditions in 20.8%. The median CCI was 5 (3–6).

### 3.2. Procedural Characteristics

In terms of procedures, 48.4% (*n* = 214) of the population underwent laparoscopic surgery compared to 51.6% (*n* = 228) that had robotic-assisted surgery (Table 2). SG was performed in 78.5% and RYGB in 21.5%. The mean duration was 104.3 ± 40.0 min.

### 3.3. Postoperative Parameters

Postoperatively, 36.0% had postoperative nausea/vomiting (PONV), 72.2% were admitted to the telemetry unit, and 27.8% were admitted to the surgical ICU as per protocol at our institution (all RYGB and all bariatric procedures in patients with BMI > 50 kg/m^2^). The hospital LOS was 34.7 ± 17.8 h.

Mean postoperative opioid consumption for the overall cohort was 11.8 ± 12.4 (95% CI: 10.6–13.0) MME. Patients undergoing laparoscopic procedures had lower PORs (10.1 ± 11.2 MME; 95% CI: 8.6–11.6) compared with those undergoing robotic procedures (13.3 ± 13.2 MME; 95% CI: 11.6–15.0) (*p* < 0.01). In the unadjusted analysis, PORs were not significantly different between SG and RYGB patients (*p* = 0.082).

Mean postoperative opioid consumption for the SG cohort was 12.0 ± 11.8 (95% CI: 10.8–13.2) MME. Patients undergoing laparoscopic SG had lower PORs (9.2 ± 9.6 MME; 95% CI: 7.8–10.6) compared with those undergoing robotic SG (14.8 ± 13.3 MME; 95% CI: 12.8–16.8) (*p* < 0.001).

Mean postoperative opioid consumption for the RYGB cohort was 9.6 ± 12.2 (95% CI: 7.1–12.1) MME. Patients undergoing laparoscopic RYGB had comparable opioid requirements (10.3 ± 11.9 MME; 95% CI: 6.0–14.6) compared with those undergoing robotic RYGB (9.5 ± 12.3 MME; 95% CI: 6.5–12.5) (*p* = 0.78).

### 3.4. Multivariable Analysis

In the multivariable logistic regression analysis evaluating factors associated with high postoperative opioid use (>10 MME), robotic surgery was associated with increased odds of elevated opioid consumption compared with laparoscopic surgery (OR 1.38, 95% CI: 1.02–1.88, *p* = 0.041) (Table 3).

Psychiatric history was independently associated with higher odds of increased opioid use (OR 1.67, 95% CI: 1.11–2.51, *p* = 0.014), as was prior surgical history (OR 1.52, 95% CI: 1.03–2.24, *p* = 0.034). BMI (*p* = 0.62), type of procedure (SG vs. RYGB; *p* = 0.055) and sex (*p* = 0.39) were not associated with postoperative opioid requirements.

## 4. Discussion

In this study, we did not find any significant association between sex, BMI, type of bariatric procedure (SG vs. RYGB) and PORs. However, robotic surgery, prior surgical history, and pre-existing mental health conditions were associated with increased PORs. Preoperative psychiatric screening may be an important consideration prior to bariatric surgery to identify vulnerable patients who will need more support and intensive postoperative pain management [22,23]. As part of the protocol in our institution, all patients undergoing bariatric surgery are subject to preoperative psychiatric evaluation in an effort to optimize postoperative outcomes, prepare the patient for the post-surgery lifestyle changes required and identify any support systems they may need. This evaluation includes a structured clinical interview and a questionnaire-based assessment to identify psychiatric disorders, including depression, anxiety, personality disorders, eating disorders, and substance use. Identification of patients with depression, anxiety, or other psychiatric disorders before surgery provides an opportunity to optimize mental health, reinforce coping strategies, and individualize perioperative pain management. Although the present study cannot establish whether optimization of psychiatric comorbidities directly reduces postoperative opioid requirements, targeted preoperative interventions, multidisciplinary behavioral support, and enhanced multimodal analgesic pathways may improve pain perception, reduce opioid exposure, and facilitate postoperative recovery. Future prospective studies should evaluate whether structured preoperative psychiatric optimization translates into improved pain-related and recovery outcomes following bariatric surgery.

In addition, prior surgical history was significantly associated with increased PORs in this study. Prior surgery changes the anatomy of the peritoneal cavity and creates adhesions that may significantly affect operative time, procedural complexity, and blood loss if adhesiolysis is required. In a multicenter cohort of 1706 patients, prior abdominal operations were associated with prolonged operative time and hospital stay [24]. More recent large single-center data similarly demonstrated increased operative difficulty in patients with prior abdominal surgery, higher rates of adhesiolysis, and conversion to open surgery [25]. Patients undergoing more complex procedures or those with a history of prior abdominal surgery may experience higher perioperative analgesic needs and could benefit from anticipatory escalation of non-opioid analgesic regimens, including scheduled acetaminophen, NSAID use when not contraindicated, and regional or adjunct anesthetic techniques where appropriate. Implementation of standardized bariatric protocol pathways with strict opioid-sparing protocols and early involvement of pain management and/or behavioral health consultation should be considered for selected high-risk patients to optimize perioperative outcomes and reduce postoperative opioid exposure.

Postoperative pain and medication requirements are critical factors affecting both recovery time and overall outcomes [1,2,3]. Inpatient opioid requirement is an indicator of the need for opioid prescription at discharge, which may increase the risk of chronic opioid use [1,2,26]. Pain experiences vary widely among patients, driven by patient-specific characteristics and perioperative factors [11,27]. Patient-related psychological factors affect pain perception and response to treatment and include pre-existing chronic pain, psychological health, age, sex, and patient expectations [28,29,30]. Among these factors, pre-existing mental health conditions, such as anxiety, depression or substance use disorder, may play an important role in postoperative opioid requirements across various types of surgery [30,31]. Patients with these conditions often have higher pain sensitivity and altered pain perception, which are associated with an increased need for opioid and non-opioid analgesics [30,32]. Anxiety and depression are associated with catastrophizing, a cognitive distortion that amplifies pain perception and may increase the need for analgesics [14,33]. Aceto et al. reported that anxious and depressed patients had higher levels of pain and showed a significant association between preoperative depression, anxiety, or alexithymia and the amount of patient-controlled opioid requests within 36 h after surgery [30]. Tapar et al. reported a strong association between the amount of morphine needed within 24 h and distress tolerance, anxiety, depression, and pain catastrophizing [34]. Overall, preoperative identification to facilitate postoperative psychological support and multimodal analgesia with pharmacologic and non-pharmacological approaches are recommended to address the needs of these patients [32,35].

Both robotic and laparoscopic bariatric procedures are associated with excellent recovery outcomes and well-established reduced PORs compared to open surgery [36]. However, the precise differences in PORs between these two approaches remain an area of ongoing research and investigation. In this study, robotic procedures (51.6%) had higher POR than laparoscopic procedures (13.3 ± 13.2 versus 10.1 ± 11.2 MME; *p* < 0.01). Interestingly, this difference was mainly driven by the population undergoing SG (14.8 ± 13.3 versus 9.2 ± 9.6 MME, respectively; *p* < 0.01). The observed difference in MME between robotic and laparoscopic SG groups reached statistical significance but the absolute difference (5.6 MME) is modest. This magnitude corresponds to roughly one to two standard postoperative morphine doses (2–4 mg) used as needed in our bariatric analgesia protocol, suggesting limited clinical impact and should be interpreted with caution. A study investigating the outcomes during the first year of a robotic bariatric program found that operative duration decreased significantly over time and that the surgeon’s learning curve stabilized after approximately 30 cases, highlighting the impact of procedural experience on perioperative outcomes [37]. A systematic review of studies reporting on the learning curves for adoption of robotic bariatric surgery reported statistically significant improvement in outcomes after the learning curve [38]. The learning curve for the entire surgical team, procedure volume, and operating room workflows affect the efficiency of robotic surgery [39]. The findings of our study may reflect the learning curve of robotic surgery at our institution. Studies comparing postoperative pain between robotic and laparoscopic techniques in bariatric surgery have yielded mixed results. Joselyn et al. conducted a retrospective analysis of 28 severely obese patients undergoing SG and showed a significant reduction in intraoperative opioid use with the robotic approach, but no significant differences in terms of PORs within the first 72 h and no difference in postoperative pain [40]. Similarly, a larger retrospective study involving 368 patients (268 laparoscopic and 82 robotic SG) found no significant differences in inpatient opioid use or pain scores [41]. Barajas-Gamboa et al. conducted a retrospective study of 484 patients undergoing SG (435 laparoscopic, 49 robotic) and reported lower pain scores over the first 24 h after surgery and significantly lower opioid requirements with robotic surgery [42]. A large database analysis of 84,034 SG patients and 36,039 RYGB patients showed no difference in SG patients but a higher rate of opioid use in the robotic RYGB group [43]. Dudash et al. investigated postoperative pain in 573 patients undergoing robotic or laparoscopic RYGB and reported comparable inpatient pain scores, opioid use, and outpatient opioid prescriptions, but the robotic RYGB group had a higher frequency of pain during the two-week follow-up [36]. Initially, it was hypothesized that robotic surgery could lead to reduced postoperative pain due to its enhanced precision, potentially minimizing tissue trauma and post-surgical complications, but there is no strong evidence supporting this hypothesis [36,40,41,42,43].

This study has several limitations. First, its retrospective single-center design introduces the potential for selection bias, information bias, and residual confounding despite multivariable adjustment. The choice of robotic versus laparoscopic approach was based on surgeon preference, patient characteristics, and institutional practice rather than randomization; therefore, confounding by indication cannot be completely excluded. Although multivariable regression was performed to adjust for measured covariates, unmeasured factors such as surgeon experience, operative complexity, intraoperative analgesic administration, protocol adherence, and temporal changes in perioperative practice may have influenced postoperative opioid requirements. The lack of association between BMI and opioid use should be interpreted cautiously, considering the narrow BMI distribution in bariatric populations. ICU admission may influence postoperative analgesic practices and opioid administration patterns. However, within our institutional bariatric protocol, ICU admission is routinely determined by procedure type (all RYGB) and BMI criteria (>50 kg/m^2^), rather than postoperative clinical deterioration or perceived illness severity. This protocol-driven allocation reduces the likelihood that ICU admission reflects differences in baseline clinical status. Second, baseline differences between the robotic and laparoscopic cohorts, including differences in procedure type, prevalence of hiatal hernia, and sex distribution, may have contributed to the observed differences in postoperative opioid consumption. Although adjusted analyses were performed, more advanced causal inference techniques, such as propensity score matching or inverse probability of treatment weighting, could further reduce treatment-selection bias and should be considered in future investigations. We elected not to conduct a post hoc analysis that was not prespecified in our original study design. Third, the observed differences may partially reflect the institutional learning curve associated with the adoption of the robotic platform rather than the surgical approach itself. Longer operative duration in the robotic cohort supports this possibility and may represent differences in case complexity, operating room workflow, or surgeon experience. Fourth, postoperative opioid consumption was limited to inpatient rescue opioid use (“as needed”) during the first 48 h after surgery. All patients were discharged according to our institution’s standardized opioid-free discharge protocol, and no patient received an opioid prescription at the time of hospital discharge. However, outpatient opioid prescriptions, opioid refill rates, emergency department visits, and long-term opioid use were not evaluated. Heterogeneity introduced by discharge timing may have introduced additional bias. Consequently, inpatient opioid consumption may not fully reflect the overall postoperative pain experience or subsequent opioid utilization. Granular data on outpatient opioid exposure prior to surgery were not consistently available in our dataset, while other relevant chronic pain conditions, such as chronic musculoskeletal pain syndromes, migraine, and fibromyalgia, may have influenced opioid requirements. Finally, despite including a relatively large cohort from a community hospital, the findings may not be generalizable to other institutions with different patient populations, perioperative analgesic protocols, surgeon experience, or stages of robotic program implementation. Prospective multicenter studies using standardized perioperative pathways and robust methods to address treatment-selection bias are needed to validate these findings and better define the independent effect of surgical platform on postoperative opioid requirements.

## 5. Conclusions

Psychiatric conditions, prior surgical history and robotic approach are associated with increased PORs following bariatric surgery, highlighting the importance of preoperative psychiatric evaluation and individualized perioperative pain management. Higher inpatient PORs were observed among patients undergoing robotic sleeve gastrectomy, while there was no significant difference in the RYGB group. Implementation of multimodal analgesia and appropriate psychological evaluation and support remain essential to optimize postoperative recovery and minimize opioid exposure. Further prospective studies are needed to better define pain outcomes across different bariatric procedures and phases of robotic adoption.

## Figures and Tables

**Table 1:** Baseline characteristics.

Variables	Total (*n* = 442)	Laparoscopic (*n* = 214)	Robotic (*n* = 228)	*p*-Value
Age (Mean (SD))	42.1 (12.0)	43.4 (11.7)	41.8 (12.3)	0.18
BMI (Mean (SD))	43.3 (7.4)	42.9 (6.2)	43.7 (8.3)	0.32
Sex (N Female (%))	383 (86.7)	174 (81.3)	209 (91.7)	0.001
Prior bariatric surgery	58 (13.1)	23 (10.7)	35 (15.3)	0.14
*Comorbidities (N (%))*				
Diabetes mellitus	165 (37.3)	83 (38.8)	82 (36.0)	0.56
Hypertension	207 (46.8)	93 (43.5)	114 (50.0)	0.18
Asthma	121 (27.4)	49 (22.9)	72 (31.6)	0.03
Obstructive sleep apnea	356 (80.5)	170 (79.4)	186 (81.6)	0.58
Gastroesophageal reflux	137 (31.0)	56 (26.2)	81 (35.5)	0.03
Hiatal hernia	133 (30.1)	48 (22.4)	85 (37.3)	<0.001
Helicobacter pylori+	64 (14.5)	29 (13.6)	35 (15.4)	0.59
Osteoarthritis	55 (12.4)	25 (11.7)	30 (13.2)	0.62
CCI (Median, Q1–Q3)	5 (3–6)	5 (3–6)	5 (3–7)	0.41
*Psychiatric medications*	92 (20.8)	49 (22.9)	43 (18.9)	0.28
Depression	74 (16.7)	31 (14.5)	43 (18.9)	0.21
Anxiety	49 (11.1)	23 (10.7)	26 (11.4)	0.82
Bipolar disorder	15 (3.4)	6 (2.8)	9 (3.9)	0.52

CCI: Charlson Comorbidity Index; BMI: body mass index; N: number; SD: standard deviation; Q1–Q3: interquartile range (first to third quartiles).

**Table 2:** Intraoperative/perioperative parameters.

Variables	Total (*n* = 442)	Laparoscopic (*n* = 214)	Robotic (*n* = 228)	*p*-Value
*Procedure*				<0.001
SG	347 (78.5)	184 (86.0)	163 (71.5)	
RYGB	95 (21.5)	30 (14.0)	65 (28.5)	
Duration (min)	104.3 (40.0)	85.5 (29.4)	119.9 (40.9)	<0.001
*Postoperative location*				<0.001
Telemetry (Step-down)	319 (72.2)	171 (79.9)	148 (64.9)	
ICU	123 (27.8)	43 (20.1)	80 (35.1)	
Hospital LOS (h)	34.7 (17.8)	34.7 (18.4)	34.6 (17.3)	0.92
PONV	159 (36.0)	77 (36.0)	83 (36.4)	0.93
*Total MME*	11.8 (12.4)	10.1 (11.2)	13.3 (13.2)	<0.01
SG	12.0 (11.8)	9.2 (9.6)	14.8 (13.3)	<0.001
RYGB	9.6 (12.2)	10.3 (11.9)	9.5 (12.3)	0.78

MME: morphine milligram equivalents; ICU: intensive care unit; SG: sleeve gastrectomy; RYGB: Roux-en-Y gastric bypass; LOS: length of stay; PONV: postoperative nausea/vomiting; min: minutes; h: hours.

**Table 3:** Multivariable logistic regression model evaluating factors associated with high inpatient postoperative opioid requirements (>10 MME).

Variable	Adjusted OR	95% CI	*p*
Platform (Robotic vs. Laparoscopic)	1.38	1.02–1.88	0.041
Type of procedure (SG vs. RYGB)	1.89	0.99–3.57	0.055
BMI	1.01	0.98–1.03	0.62
Sex (male)	1.21	0.78–1.88	0.39
Psychiatric history	1.67	1.11–2.51	0.014
Prior surgical history	1.52	1.03–2.24	0.034

OR: odds ratio; CI: confidence interval; *p*: *p*-value; BMI: body mass index; SG: sleeve gastrectomy; RYGB: Roux-en-Y gastric bypass.

## Data Availability

The data of this study are included in the article and summarized in tables. Further requests can be directed to the authors and data are available upon reasonable request.
